# The Effect of Collagen Membrane Fixation with Pins on Buccal Bone Regeneration in Immediate Dental Implant Sites: A Preclinical Study in Dogs

**DOI:** 10.3390/jfb16080281

**Published:** 2025-07-31

**Authors:** Yuma Hazama, Takahisa Iida, Niklaus P. Lang, Fernando M. Muñoz Guzon, Giovanna Iezzi, Daniele Botticelli, Shunsuke Baba

**Affiliations:** 1 Department of Oral Implantology, School of Dentistry, Osaka Dental University, 8-1 Kuzuhahanazonocho, Hirakata, Osaka 573-1121, Japan; y.hazama.d@gmail.com (Y.H.); iidataka@iris.ocn.ne.jp (T.I.); baba-s@cc.osaka-dent.ac.jp (S.B.); 2 ARDEC Academy, 47923 Rimini, Italy; 3 Department of Periodontology, University of Berne, 3010 Bern, Switzerland; nplanggalen@gmail.com; 4 Ibonelab, Centro de Empresas e Innovación Nodus, 27003 Lugo, Spain; fernandom.munoz@usc.es; 5 Department of Veterinary Clinical Sciences, Facultade de Veterinaria, Terra Campus, Universidade de Santiago de Compostela, 27002 Lugo, Spain; 6 Department of Medical, Oral and Biotechnological Sciences, University of Chieti-Pescara, 66013 Chieti, Italy; gio.iezzi@unich.it

**Keywords:** biocompatible materials, bone transplantation, bone substitutes, histological techniques, surgical procedures, operative, tooth extraction, bone regeneration, membranes, artificial + collagen

## Abstract

Background: The role of collagen membrane fixation during guided bone regeneration (GBR) remains debatable, particularly in post-extraction sockets with buccal defects and concomitant immediate implant placement. This study evaluated whether or not fixation with titanium pins improved regenerative outcomes. Methods: Six adult Beagle dogs received bilateral extractions of the fourth mandibular premolars. An implant was immediately placed in both the distal alveoli, and standardized buccal bone defects (5 mm height, 3–2 mm width) were prepared. All defects were filled with a slowly resorbing equine xenograft and covered by a resorbable pericardium membrane. At the test sites, the membrane was apically fixed with pins, while no fixation was applied to the control sites. After 3 months of healing, histomorphometric analyses were performed. Results: The vertical bone gain of the buccal crest was 3.2 mm in the test sites (pin group) and 2.9 mm in the control sites (no-pin) (*p* > 0.754). No significant difference was found in terms of bone-to-implant contact (BIC). However, residual graft particles were located significantly more coronally in the pin group compared to the no-pin group (*p* = 0.021). Morphometric analyses revealed similar new bone formation within the groups, but with higher amounts of residual xenograft and soft tissue in the pin group. Conclusions: Membrane fixation did not significantly enhance vertical bone gain, and although the slightly higher regeneration in the pin group (3.2 mm vs. 2.9 mm) may hold clinical relevance in esthetically sensitive areas and osseointegration, it appeared to limit apical migration of the grafting material.

## 1. Introduction

Guided bone regeneration (GBR) has become a popular technique in implant dentistry, particularly when addressing alveolar bone defects to facilitate proper implant placement and long-term stability [1,2,3]. The technique relies on the placement of a barrier membrane to exclude soft tissue proliferation and to maintain space for osteogenic cell proliferation within the defect area [4,5,6]. Among the various types of membranes available, resorbable collagen membranes are widely adopted due to their biocompatibility, hemostatic properties, and the absence of a need for removal surgery [4,7,8,9]. However, despite their clinical popularity, several aspects of their application remain unclear, particularly the necessity and clinical benefit of membrane fixation. Several studies have reported that substantial bone resorption occurs during the first weeks after tooth extraction, particularly at the buccal aspect, which underscores the importance of early stabilization in GBR procedures [10,11].

A recent comprehensive review [12] underscored that while collagen membranes offer promising results in GBR, their mechanical behavior, cross-linking properties, and interaction with graft materials may influence their clinical performance. Furthermore, the need for membrane fixation should be assessed in the light of the defect morphology, surgical technique, and selection of materials. A preclinical study [13] provided evidence that membrane fixation may enhance the expression of osteogenic markers such as BMP-2, FGF-2, VEGF, and osteocalcin, leading to significantly greater new bone formation compared to non-fixed membranes.

Conversely, other studies have shown successful dimensional stability of the regenerated bone, even in the absence of membrane fixation. In a canine study [14], membrane fixation did not significantly impact the total augmented bone volume in contained defects, although it increased the width of the ridge crest when using specific membrane types. Moreover, a retrospective clinical study showed that GBR procedures for both vertical and horizontal bone volume augmentation achieved clinically relevant outcomes without membrane fixation, using a retentive flap design to stabilize the augmented sites [15]. However, these results indicated a more pronounced reduction in horizontal ridge width over time, raising the question of whether or not certain defect configurations, such as uncontained or vestibular defects, may be more susceptible to bone volume loss without additional membrane stabilization.

From a clinical and surgical perspective, fixation of membranes with pins or screws is often associated with increased operative time and the potential need for a second intervention to remove the fixation devices. To address these limitations, an alternative technique using periosteal vertical mattress sutures to stabilize the membrane and graft material was proposed, avoiding the complications associated with rigid fixation while aiming to maintain stability of the regenerative complex [16].

Despite a growing body of evidence, there remains a gap in the literature regarding the behavior of collagen membranes in vestibular bone defects in post-extraction sockets receiving immediate implants. This may be a scenario in which membrane displacement and graft destabilization are potential risks due to the lack of natural bony containment. Therefore, this preclinical study aimed to assess whether membrane fixation with pins improves graft stability and bone regeneration in buccal defects associated with immediate implant placement.

## 2. Materials and Methods

### 2.1. Ethical Statement

All procedures involving animals were carried out in accordance with the principles established by Directive 2010/63/EU, which governs the protection of animals used for scientific experimentation. The research protocol was reviewed and approved by both the Ethical Committee of the Rof Codina Foundation and the relevant regional authority (Xunta de Galicia, Xefatura Territorial da Consellería do Medio Rural, Ronda da Muralla 70, Lugo, Spain), under the authorization code 01/20/LU-001 of 8 April 2020. This study was also designed and conducted in accordance with the ARRIVE (Animal Research: Reporting of In Vivo Experiments) guidelines to ensure transparency and reproducibility.

### 2.2. Experimental Animals

The experiment included six female Beagle dogs, each approximately 6 years of age and weighing an average of 11.5 kg. The inclusion criteria required the animals to be healthy, with no systemic diseases or oral pathologies. The animals were sourced from Isoquimen (Barcelona, Spain) in May 2023 and underwent a quarantine period of three weeks prior to any surgical intervention, which was carried out in June 2023. All animal handling, housing, and experimental procedures were carried out at the CeBioVet research facility in Lugo, Spain.

### 2.3. Experimental Design

**Figure 1:**
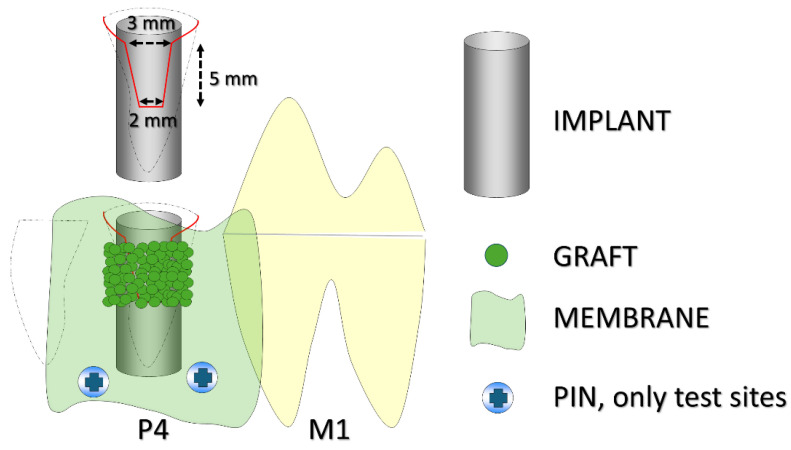
A schematic illustration of the experimental design. The black dashed lines indicate the profile of the alveoli, while the red line outlines the buccal defect. P4 refers to the fourth premolar and M1 to the first molar. The implant was placed in the distal alveolus of P4. Pins were used only at the test sites.

The fourth mandibular premolars were extracted bilaterally, and implants were immediately placed into the distal alveoli, with the implant shoulder positioned at the level of the buccal bony crest. Standardized buccal bony defects were prepared on both sides, measuring about 5 mm in height, with a coronal width of approximately 3 mm and an apical width of about 2 mm. Both defects were filled with a xenograft protected by a collagen membrane. At the test sites, the membranes were fixed apically with pins, while no fixation was applied at the control sites (Figure 1).

### 2.4. Sample Size

Sample size calculation was based on data from a previous dog study [17], in which a similar defect model was employed without membrane fixation. In that study, the use of a low-resorbable xenograft resulted in a vertical bone gain of 4.3 ± 0.7 mm at a 6 mm-high buccal defect. It was assumed that the application of 2 fixation pins in conjunction with a smaller buccal defect could result in an additional 1 mm of vertical bone gain. This threshold was considered clinically relevant, as even minor improvements in buccal bone height may contribute to enhanced soft tissue support and long-term positive esthetic outcomes in implant therapy. A standard deviation of 0.7 mm was used, directly derived from the variability reported in that study, and reflects biological differences in bone regeneration under controlled experimental conditions. Based on these parameters, a Type I error probability (α) of 0.05 and a statistical power (1−β) of 0.8, a total of 6 subject pairs was required to detect a significant difference between groups (PS Power and Sample Size Calculations, Version 3.0) [18,19].

### 2.5. Randomization, Allocation Concealment, and Blinding Procedures

One of the authors (D.B.), who had no role in the surgical procedures, conducted the randomization using an electronic system. Treatment allocations were placed in sealed, opaque envelopes, were coded and stored by the same author, and were only revealed after implant placement and the preparation of the buccal defect in each animal. Histological slides were anonymized to ensure blinding during microscopic evaluation. However, the presence of fixation pins in some samples partially compromised the blinding. This limitation is acknowledged, and its potential bias was minimized by the use of standardized measurement protocols. However, in a limited number of cases, the presence of a fixation pin within the histological section made it possible to infer the treatment group, partially compromising observer blinding.

### 2.6. Biomaterials

Calcitos^®^ (Bioteck^®^ S.p.A., Arcugnano, Vicenza, Italy) is a slowly resorbing bone grafting material obtained from equine cancellous bone. It is processed at high temperatures to eliminate organic components, yielding a hydroxyapatite-rich matrix that closely resembles human bone in both structural and chemical profiles.

Heart^®^ (Bioteck^®^ S.p.A., Arcugnano, Vicenza, Italy) is a resorbable membrane produced from equine pericardium. Through an enzymatic de-antigenation process, antigenic elements are removed while preserving the native architecture of collagen and elastin fibers, maintaining the membrane’s original three-dimensional structure.

### 2.7. Anesthesia Procedures

Animals received premedication via intramuscular injection with a combination of medetomidine (10 µg/kg; Sededorm^®^ 1 mg/mL, Vetpharma Animal Health, Barcelona, Spain) and morphine (0.3 mg/kg; Morfina Braun 2%, B. Braun Medical, Barcelona, Spain). General anesthesia was induced intravenously with propofol (2 mg/kg; Propofol Lipuro^®^ 10 mg/mL, B. Braun, Melsungen, Germany) and maintained using isoflurane (1–1.5%) in oxygen (Vetflurane^®^ 1000 mg/g, Virbac SA, Carros, France). Throughout the procedure, physiological parameters, including heart and respiratory rates, arterial pressure, and end-tidal CO_2_, were monitored continuously. Recordings were performed every 5 min during the induction phase and subsequently at 15 min intervals.

### 2.8. Surgical Procedures

**Figure 2:**
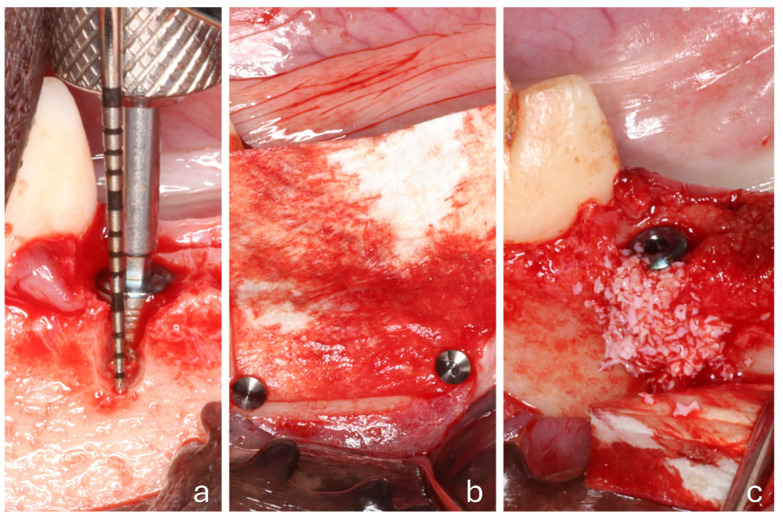
A clinical view of a test site. (**a**) The placement of the implant within the extraction socket and the creation of a buccal defect; (**b**) the collagen membrane positioned and fixed with 2 pins; (**c**) the membrane displaced to facilitate the placement of the biomaterial.

All surgical procedures were performed by an experienced oral surgeon (T.I.). A full-thickness mucoperiosteal flap was raised, and the fourth premolars were extracted bilaterally. Implants (3.5/10 mm; I-ON Implant Conical Platform; AB Dental Devices Ltd., Ashdod, Israel) were placed in the distal sockets of both sites, with the implant shoulder positioned at the level of the buccal bony crest. A cover screw was attached to each implant. Standardized buccal bony defects were prepared on both sides, measuring 5 mm in height, with an approximate width of 3 mm coronally and 2 mm apically (Figure 2a). A pericardium membrane (Heart Bioteck^®^ S.p.A., Arcugnano, Vicenza, Italy) was placed in both groups. However, apical stabilization using titanium pins (Titan Pin Set; Botiss biomaterials GmbH, Zossen, Germany) was performed only at the test sites (Pin group; Figure 2b), while no pins were applied at the control sites (No-pin group). At the test sites, small holes were first prepared in the apical region using a round bur. The pins were then positioned with a dedicated pin holder and inserted into the prepared holes while simultaneously securing the membrane. Final fixation was achieved by gently tapping the pins into place using a surgical mallet.

The defects were subsequently grafted with Calcitos^®^ (Bioteck^®^ S.p.A., Arcugnano, Vicenza, Italy) (Figure 2c). Following slight flap mobilization, interrupted sutures (Vicryl 4-0) were placed to achieve complete primary closure, leading to submerged healing.

### 2.9. Housing and Husbandry

Wound care was maintained by gently cleaning the surgical regions three times per week using gauze soaked in 0.12% chlorhexidine during the first two weeks. Thereafter, chlorhexidine gel 1% was applied regularly until study completion. Postoperative analgesia included intramuscular buprenorphine (0.01 mg/kg every 8 h; Bupaq, Richter Pharma AG, Wels, Austria). Meloxicam (Loxicom, Norbrook Laboratories, Monaghan, Ireland) was administered intravenously at 0.2 mg/kg before surgery, followed by oral dosing at 0.1 mg/kg once daily for two days. To minimize the risk of infection, antibiotics were administered: cefazolin (20 mg/kg IV prior to surgery; Cefazolina Normon, Madrid, Spain) and a single subcutaneous dose of cefovecin (8 mg/kg; Convenia, Zoetis, Madrid, Spain) immediately after the procedure.

### 2.10. Euthanasia

Three months after the final surgical intervention, the animals were sedated and euthanized through intravenous injection of sodium pentobarbital at a dosage of 200 mg/kg (Dolethal, Vetoquinol, France). The specimens were harvested and stored in 10% buffered formalin under refrigeration for at least one week prior to histological processing.

### 2.11. Histological Preparation

Following fixation, the samples underwent histological processing using the Precise 1 Automated System (Assing, Rome, Italy). Dehydration was performed through an ascending series of alcohols, following which the specimens were embedded in Technovit 7200 VLC (Kulzer, Wehrheim, Germany), a glycol methacrylate resin. Once polymerized, the blocks were sectioned longitudinally in the buccolingual plane using a high-precision diamond saw to a thickness of approximately 150 µm, then thinned to ~30 µm using a grinding unit. Sections aligned with the implant’s central axis were stained with acid fuchsin and toluidine blue. Orientation was verified based on anatomical landmarks and the implant-healing cap junction to ensure consistent identification across all samples.

### 2.12. Clinical Parameters

Immediately prior to implant placement, the buccal bony crest width was measured 1 mm apically to its most coronal point using an Iwanson caliper (Koiné Italia snc, Milano, Italy). A UNC periodontal probe (Hu-Friedy Italy Srl, Milan, Italy) was used to record the horizontal buccolingual width of the socket and the relative positions of the buccal and lingual bony margins in relation to the implant shoulder. The insertion torque was documented at the time of implant placement using a mechanical wrench. After implant placement, the horizontal and vertical dimensions of the residual bony buccal gap were measured before the preparation of the standardized buccal defect.

### 2.13. Histological Evaluations

Histological analysis was performed using a Nikon Eclipse microscope (Nikon Corporation, Tokyo, Japan) connected to a digital imaging system (NIS-Elements D 5.11.00; Laboratory Imaging, Nikon Corporation, Tokyo, Japan), which was used for both linear and morphometric evaluations.

The following landmarks were used for histological measurements (Figure 2): IS (implant shoulder), C (most coronal point of newly formed bone), B (BIC; most coronal point of bone-to-implant contact), and X (most coronal level of residual xenograft granules). Vertical distances from IS to C, B, and X were assessed. The height of the regenerated bone was calculated relative to the original vertical bony defect of 5 mm.

**Figure 3:**
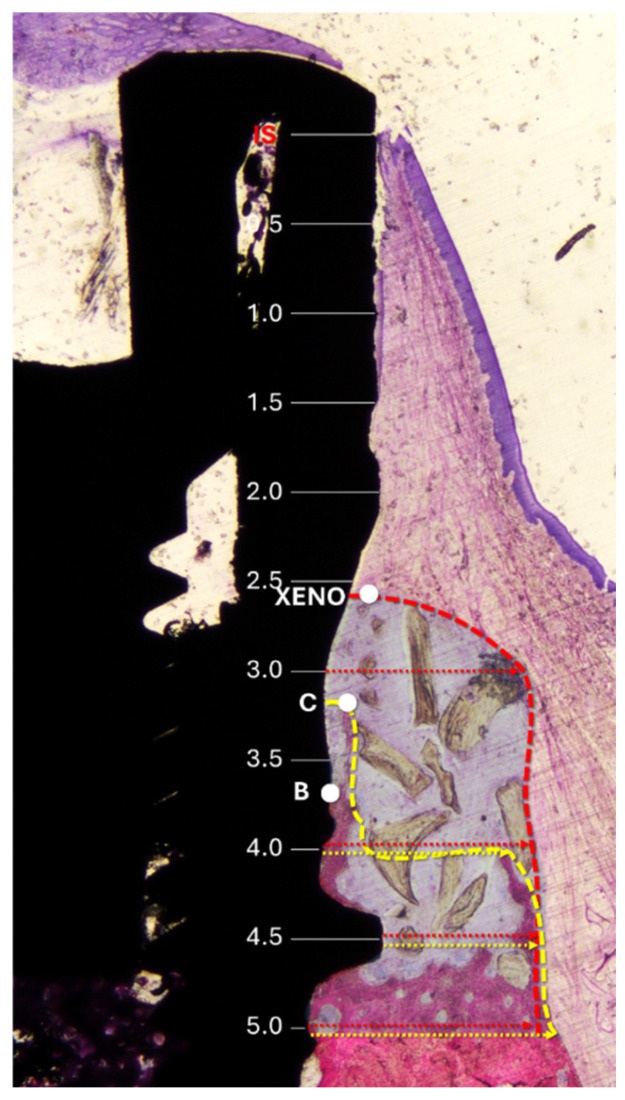
Histological references and measurements. IS, implant shoulder; C, top of the bone crest at the lingual aspect; B, coronal level of osseointegration. Distances from the implant surface to the most lateral extension of the bone (yellow arrows) and xenograft (red arrows) were measured at 0.5 mm intervals from IS to 5 mm apical to IS. Dashed lines delineate the periphery of the bone (yellow) and the xenograft (red). The region of interest is marked with a translucent overlay to allow for clear visualization of the underlying histological structures.

Horizontal measurements were taken from the implant surface to the lateral margin of the new bone and the xenograft material at 0.5 mm intervals, starting from the implant shoulder and extending apically to 5 mm (Figure 3).

In addition, morphometric analysis was carried out within a region delineated by the implant surface and extending from the most coronal and lateral hard tissue to 5 mm apically to the IS (Figure 3). A point-counting method was applied by overlaying a grid on the histological sections at the microscope/computer unit. The amount of each tissue was reported as a percentage relative to the total number of evaluated points. In addition, the percentage values were converted into absolute measurements (mm^2^), based on the total area analyzed.

### 2.14. Data Analysis

Histological evaluation was carried out using a calibrated examiner (E.F.D.R.; see acknowledgments) under the continuous supervision of a senior researcher (D.B.). The primary variables analyzed were buccal IS-C, IS-B, and IS-X. Data distribution was assessed using the Shapiro–Wilk test for normality. Based on the outcome, either a paired Student’s *t*-test or the Wilcoxon signed-rank test was employed for comparing groups. Statistical significance was determined at a threshold of α = 0.05. The effect size (Cohen’s d for paired samples) and 95% confidence intervals were calculated for the primary outcome variables. All analyses were performed using GraphPad Prism (version 10.5; GraphPad Software, San Diego, CA, USA).

## 3. Results

No complications were observed during the healing period, and all histological samples were successfully processed and analyzed, ensuring a final sample size of n = 6.

### 3.1. Clinical Evaluation

The coronal buccolingual dimension of both alveoli was wider than the implant diameter, and a buccal gap < 1 mm resulted (Table 1).

All implants were inserted with an initial torque with a mean value around 40 Ncm. No statistically significant differences were disclosed.

### 3.2. Histological Qualitative Analysis

**Figure 4:**
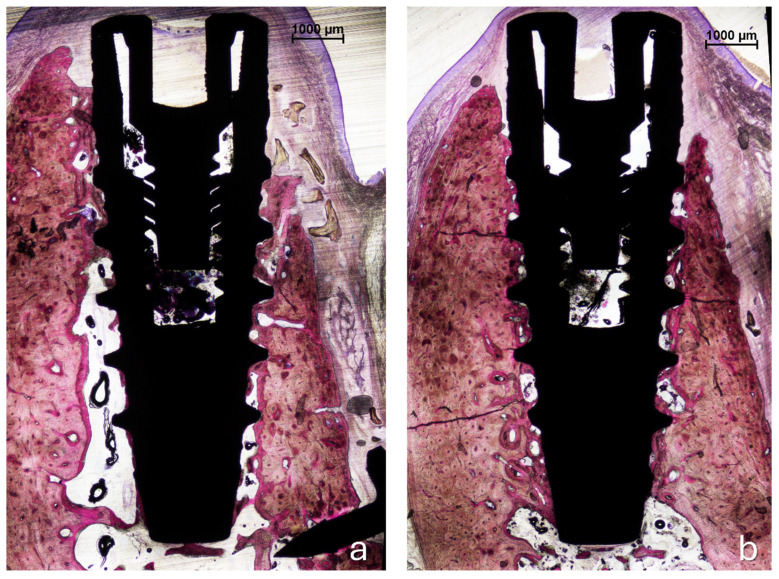
Photomicrographs of ground sections. (**a**), The pin site; (**b**), the no-pin site. Bone regeneration was evident in all samples after 3 months of healing, with new bone surrounding the implant body in both the trabecular and cortical regions. Acid fuchsin and toluidine blue stain. The bar scale is included in the image.

**Figure 5:**
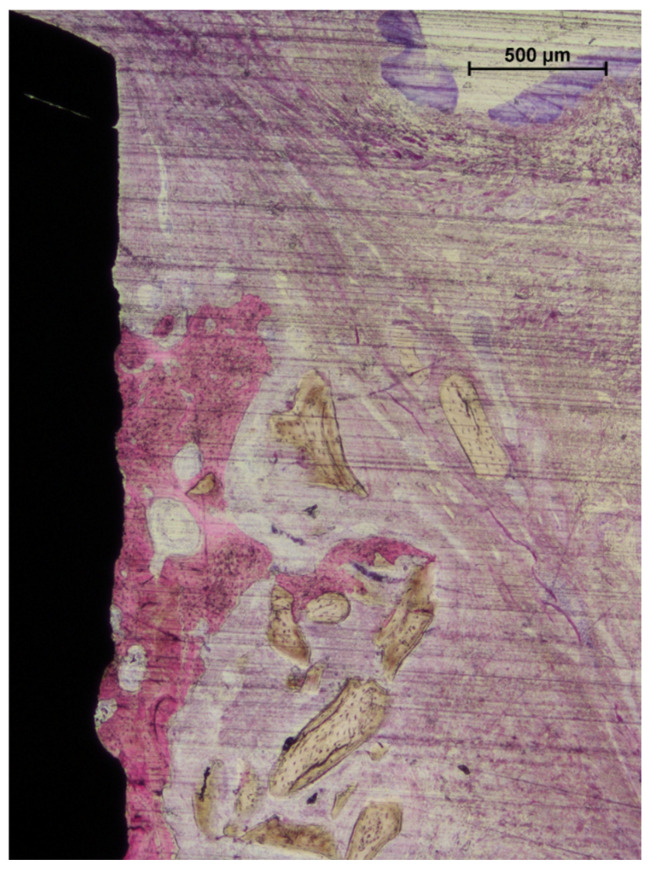
A photomicrograph of a ground section. In some cases, bone regeneration extended up to areas near the implant shoulder. Beyond the regenerated bone, residual graft granules were observed to be embedded within connective tissue. Acid fuchsin and toluidine blue stain. The bar scale is included in the image.

All histological sections were successfully collected, resulting in a complete dataset (n = 6). In the no-pin group, one implant exhibited mucosal perforation; however, no associated inflammatory cell infiltration was observed. Bone regeneration was evident in all samples in both groups (Figure 4a,b). The amount of residual xenograft appeared greater in the pin group, while several specimens in the no-pin group showed minimal or no remaining granules. When present, the graft particles were traversed by newly formed bone, occasionally reaching the coronal edge of the augmented region (Figure 4b and Figure 5).

**Figure 6:**
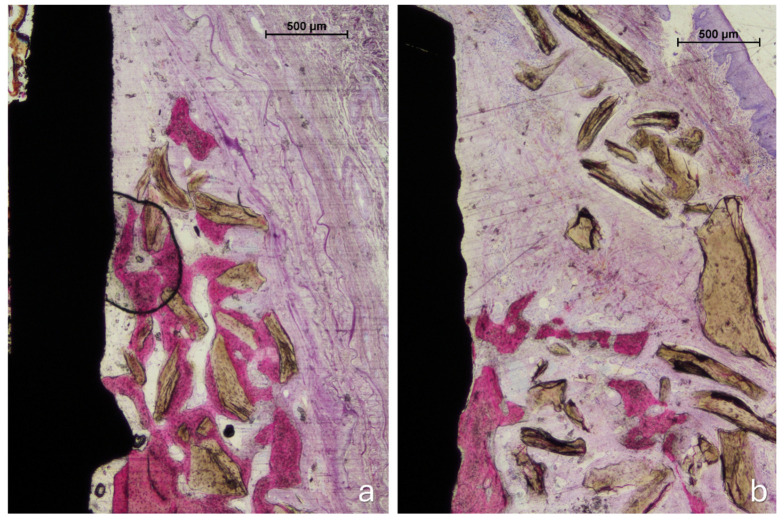
Photomicrographs of ground sections. (**a**) The pin site: new bone regenerated from the edges of the defect and integrated with adjacent graft particles. The regenerated tissue partially encapsulated the grafted area, though not its outermost portions. (**b**) The no-pin site: the coronal region was occupied exclusively by biomaterial granules embedded in connective tissue. Staining: acid fuchsin and toluidine blue. The bar scale is included in the image.

Signs of early graft integration were observed in several augmented areas. However, the outermost zones of the grafted regions frequently lacked new bone formation (Figure 6a,b).

### 3.3. Histological Quantitative Analyses

The vertical distance from the implant shoulder to the most coronal point of the newly formed bone (IS–C) was 2.1 ± 1.0 mm in the no-pin group, corresponding to a vertical bone gain of 2.9 mm (Table 2). In the pin group, IS–C measured 1.8 ± 1.1 mm, indicating a gain of 3.2 mm. The effect size between the pin and no-pin groups was negligible and not statistically significant (Cohen’s d = −0.14; 95% CI: −1.19 to 0.92; *p* = 0.754, paired *t*-test).

The IS–B distance (implant shoulder to the most coronal point of bone-to-implant contact) was 2.9 ± 0.8 mm in the no-pin group and 2.3 ± 1.1 mm in the pin group. The effect size between the pin and no-pin groups was small and not statistically significant (Cohen’s d = −0.34; 95% CI: −1.42 to 0.74; *p* = 0.443, paired *t*-test).

The position of the most coronal xenograft granules (Xeno) differed significantly between groups: in the no-pin group, Xeno was located 4.3 ± 1.4 mm from the IS, while in the pin group it was 1.5 ± 1.7 mm. The effect size between the pin and no-pin groups in IS-X was large and statistically significant (Cohen’s d = −1.35; 95% CI: −2.80 to 0.10; *p* = 0.021, paired *t*-test).

**Figure 7:**
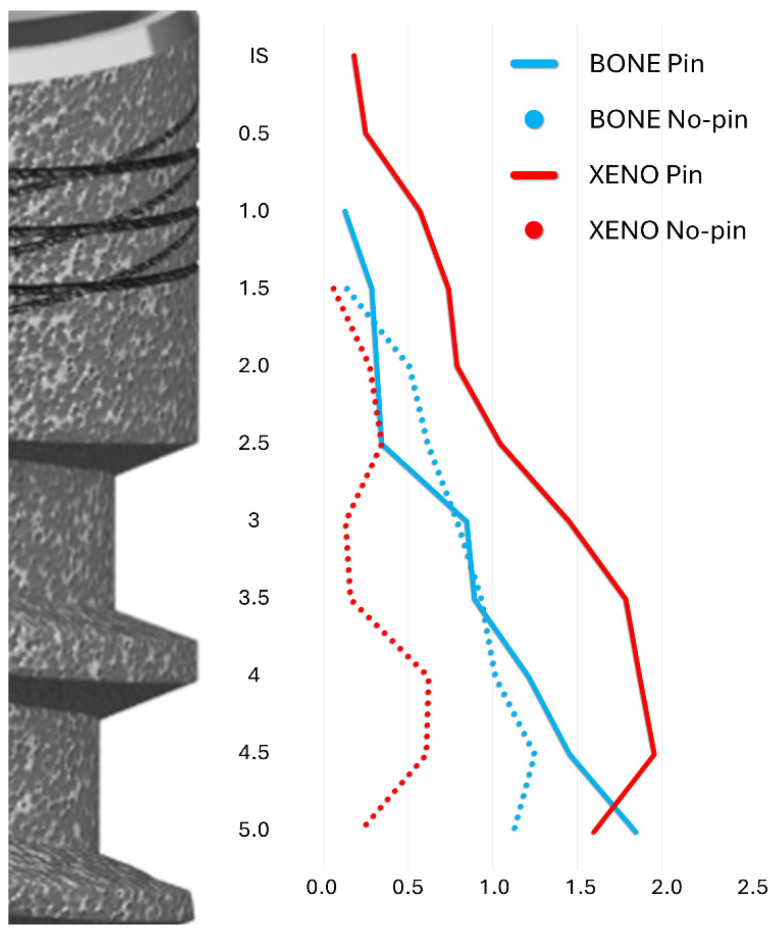
A graph illustrating the lateral extension at both the pin and no-pin sites of the bone and xenograft (Xeno) from the implant surface taken at 0.5 mm intervals from the IS to 5 mm apical to the IS. Blue lines: Bone; Red lines: Xenograft. Plain lines: Pin group; dotted lines: No-pin group.

The horizontal extension of newly formed bone was comparable between groups (Table 3). However, due to the greater presence of residual xenograft in the pin group, a wider buccal hard tissue volume was noted, although this did not reach statistical significance (Figure 7).

Histomorphometric analysis revealed similar areas of new bone formation: 1.25 ± 0.80 mm^2^ in the no-pin group and 1.28 ± 0.82 mm^2^ in the pin group (Table 4). In contrast, the area occupied by residual xenograft was minimal in the no-pin group (0.09 ± 0.16 mm^2^), while it was substantially greater in the pin group (0.73 ± 0.66 mm^2^). The soft tissue component was also more pronounced in the pin group (2.35 ± 2.08 mm^2^) compared to the no-pin group (0.37 ± 0.36 mm^2^).

## 4. Discussion

This preclinical study compared the outcomes of collagen membrane fixation versus non-fixation in standardized buccal defects treated with immediate implant placement and a xenograft. Vertical bone regeneration was similar between groups, with mean gains of 2.9 mm in the no-pin group and 3.2 mm in the pin group. Although the extent of new bone formation and bone-to-implant contact did not differ significantly, membrane fixation resulted in a more coronal and lateral localization of residual graft particles. This greater preservation of the xenograft in the Pin group contributed to increased buccal tissue volume, although the horizontal bone extension and the total new bone area remained comparable. Notably, soft tissue and residual biomaterial were more prominent in the pin group, while xenograft remnants were almost entirely absent in most no-pin specimens. These findings suggest that membrane fixation may support graft stability and volume maintenance, although it does not appear to enhance new bone formation during the early healing phase.

Membrane fixation in guided bone regeneration, whether achieved with resorbable or non-resorbable pins, is commonly used to stabilize the graft and protect the underlying defect [20,21,22]. However, this approach has potential drawbacks, such as the need for a secondary intervention to remove non-resorbable pins or the risk of surgical complications, including fracture of thin buccal plates or injury to adjacent roots. As an alternative, subperiosteal suturing techniques have been proposed to secure the membrane while avoiding rigid fixation [16].

Several studies have highlighted potential advantages of membrane fixation. In a rabbit calvarial model, An et al. observed that fixed membranes enhanced the expression of osteogenic factors and resulted in greater bone formation than non-fixed membranes [13]. A follow-up study by the same group, using collagen bone blocks and collagen membranes with or without fixation, confirmed these findings, reporting larger areas of new bone and higher levels of osteogenic markers in the fixed groups [23]. Nevertheless, a systematic review on GBR using collagen membranes and particulate grafts concluded that membrane fixation was only weakly associated with increased vertical bone gain [24].

Conversely, other studies reported different results. In a retrospective clinical study, horizontal and vertical augmentations using particulate bone grafts and resorbable membranes were performed without fixation, relying instead on a retentive flap technique [15]. Cone-beam computed tomography revealed stable vertical bone dimensions, but a reduced capacity to preserve ridge width. Similarly, in a preclinical model involving eight Beagle dogs, standardized box-shaped defects were grafted and covered with non-cross-linked collagen membranes, with or without fixation [14]. In this contained defect scenario, membrane fixation did not improve overall ridge volume, although it increased coronal ridge width depending on the membrane type.

It is worth noting that, in dog studies [14,25], the defect geometry provided intrinsic containment for the graft material. In contrast, in the present model, primary containment was minimal due to the presence of the implant within the socket. This difference may explain the pronounced graft particle loss observed in the no-pin group compared to the pin group.

The present study demonstrated a greater volume of regenerated buccal tissues in the pin group, despite only a modest difference in vertical bone regeneration: 64% in the pin group versus 58% in the no-pin group. The horizontal gain observed in the pin group may offer significant esthetic advantages, particularly when using a slowly resorbable material capable of maintaining the augmented volume over time. However, further studies that evaluate long-term data are needed to better understand the remodeling potential and esthetic performance of the graft–host interface. Despite these considerations, complete regeneration of the buccal bone wall was not achieved in the present study. Instead, the regenerated tissue exhibited a hybrid composition, consisting of newly formed bone interspersed with xenograft granules embedded in both mineralized and soft connective tissues. It should also be emphasized that the results are not consistently reproducible across all cases. While in some instances a good preservation of the crestal bone or satisfactory regeneration of buccal defects was observed, despite the presence of hybrid bone, in others, suboptimal outcomes were reported [4,26,27]. In the outermost regions, graft particles were frequently surrounded by poorly vascularized connective tissue, resembling scar-like tissue [28]. This outcome reflects a reparative process rather than a complete regenerative one. Future studies are needed to assess whether such hybrid tissue can maintain volume and esthetics or if it is prone to resorption, with newly formed bone interspersed with xenograft granules embedded both in mineralized and soft connective tissues.

From a clinical perspective, membrane fixation may offer mechanical benefits in stabilizing the graft, particularly in post-extraction sockets with minimal bony containment. However, the lack of significantly increased bone formation indicates that fixation alone is insufficient to ensure full regeneration of the buccal bone wall. Clinicians should evaluate defect morphology carefully, bearing in mind that current protocols may maintain volume without achieving the formation of fully vital and functional bone.

These findings reinforce the need for further research into simplified and predictable regenerative approaches capable of producing more complete and biologically sound outcomes, especially in anatomically challenging situations. Different combinations of graft materials and membranes may lead to variable outcomes, potentially achieving the dual objective of obtaining and maintaining an adequate volume of regenerated tissue over time, while simultaneously promoting a higher proportion of newly formed bone and a lower percentage of residual graft. Among these emerging techniques, the application of dense polytetrafluoroethylene (d-PTFE) membranes [29,30] and cortical laminae [31,32] has shown particularly promising results, offering enhanced stability and potential for improved regenerative outcomes. These approaches may favor the formation of sound, well-structured bone rather than hybrid tissue, which could potentially compromise implant osseointegration and affect long-term clinical success. In fact, several studies have demonstrated that implant placement in areas of hybrid bone may lead to extensive contact between the implant surface and residual graft particles, effectively preventing portions of the implant from establishing direct bone contact [33,34]. This lack of true osseointegration not only raises concerns regarding primary stability but may also predispose the site to future biological and mechanical complications.

This study has several limitations inherent to its preclinical design. While the animal model was appropriate for ethical and methodological reasons, the results may not be directly applicable to human clinical settings. Moreover, the short healing period might only reflect an intermediate reparative stage, and longer observation is needed to assess the complete regeneration potential. The small sample size, although justified by the 3Rs principles [35], limits the statistical power and may have hindered the detection of more nuanced differences between groups. A larger sample size would be needed to confirm these observations.

Moreover, the standardized experimental environment does not account for patient-related variables such as systemic health, individual healing capacity, and anatomical diversity, all of which strongly influence clinical outcomes. The relatively short healing period also restricts the ability to draw conclusions regarding long-term tissue remodeling and graft integration.

Despite these constraints, this study effectively achieved its primary objective: to evaluate the biological and dimensional effects of membrane fixation in a challenging post-extraction defect model. Further studies are recommended to investigate longer healing periods and to test novel combinations of materials and techniques aimed at enhancing the predictability and quality of bone regeneration.

## 5. Conclusions

While membrane fixation did not significantly improve bone gain or bone-to-implant contact, it appeared to reduce apical displacement of the graft material. In contained post-extraction defects, pin fixation may provide mechanical stabilization, although it does not seem to substantially influence the amount of newly formed bone.

Moreover, the use of a low-resorbable xenograft and a resorbable collagen membrane, whether fixed or not, did not result in complete regeneration of the buccal wall. The regenerated tissue displayed a heterogeneous composition of the new bone, connective tissue, and the residual graft material. This partial and inconsistent outcome underscores a clinical limitation of current GBR protocols in buccal defects and suggests that more effective biomaterials and adjunctive strategies may be necessary to achieve complete and functional bone reconstruction in demanding anatomical contexts. Future research should explore bioactive scaffolds, growth factor-loaded membranes, or cortical laminae that may enhance osteogenesis and support the formation of more homogeneous, mature bone.

## Figures and Tables

**Table 1:** The mean clinical values ± standard deviations reported in millimeters. B-L, horizontal buccal–lingual dimension of the alveolus; B width, buccal bone crest width; Gap, residual buccal horizontal gap; B depth, vertical buccal residual defects. No statistically significant differences were found between the two groups.

	B-L	B Width	Gap	B Width + Gap	B Depth	Torque
No-pin	4.5 ± 0.8	0.8 ± 0.2	0.7 ± 0.5	1.6 ± 0.4	1.3 ± 1.5	37.5 ± 14.1
Pin	4.5 ± 0.8	0.6 ± 0.3	0.9 ± 0.2	1.5 ± 0.7	2.8 ± 1.6	43.3 ± 4.1
p value	0.093	>0.999	>0.999	0.675	0.191	>0.999

**Table 2:** Mean histological values ± standard deviations, reported in millimeters. IS, implant shoulder; B, coronal level of osseointegration; C, top of the bone crest; Xeno, most coronal level of the xenograft. Bone gain values are indicated in parentheses. *, *p* < 0.05.

	Buccal			Lingual	
	IS-C (Gain)	IS-B (Gain)	IS-Xeno	IS-B	IS-C
No-Pin	2.1 ± 1.0 (2.9)	2.9 ± 0.8 (2.1)	4.3 ± 1.4 *	1.5 ± 0.8	0.4 ± 0.6
Pin	1.8 ± 1.1 (3.2)	2.3 ± 1.1 (2.7)	1.5 ± 1.7 *	0.8 ± 0.4	0.1 ± 0.7
*p* value	>0.754	0.443	0.021	0.110	0.347

**Table 3:** The horizontal extension at the control (-C) and test (-T) sites of the bone (BONE) and xenograft (XENO) from the implant surface.

	IS	0.5	1	1.5	2	2.5	3	3.5	4	4.5	5
BONE No-Pin	0.0 ± 0.0	0.0 ± 0.0	0.0 ± 0.0	0.1 ± 0.3	0.5 ± 0.4	0.6 ± 0.4	0.8 ± 0.4	0.9 ± 0.5	1.0 ± 0.3	1.3 ± 0.3	1.1 ± 0.3
BONE Pin	0.0 ± 0.0	0.0 ± 0.0	0.1 ± 0.2	0.3 ± 0.4	0.3 ± 0.4	0.3 ± 0.5	0.8 ± 0.6	0.9 ± 0.6	1.2 ± 0.7	1.5 ± 0.8	1.8 ± 1.0
*p* value	-	-	0.500	0.250	0.489	0.438	0.870	0.926	0.844	0.438	0.126
XENO No-Pin	0.0 ± 0.0	0.0 ± 0.0	0.0 ± 0.0	0.1 ± 0.1	0.3 ± 0.5	0.3 ± 0.5	0.1 ± 0.3	0.2 ± 0.4	0.6 ± 0.8	0.6 ± 0.8	0.2 ± 0.5
XENO Pin	0.2 ± 0.4	0.2 ± 0.6	0.6 ± 0.6	0.7 ± 0.7	0.8 ± 0.9	1.0 ± 1.0	1.5 ± 1.0	1.8 ± 1.3	1.9 ± 1.3	2.0 ± 1.4	1.6 ± 1.8
*p* value	>0.999	>0.999	0.125	0.125	0.375	0.375	0.063	0.063	0.085	0.144	0.250

**Table 4:** Morphometric analysis. The tissue contents expressed in percentages (%) and the area in mm^2^ within the coronal 5 mm from the IS.

	Bone	XENO	Soft Tissue	Area
No-Pin %	61.5 ± 33.8	3.5 ± 5.7	35.0 ± 34.1	100
Pin %	34.5 ± 22.0	15.1 ± 7.5	50.3 ± 17.4	100
*p* value	0.156	0.094	0.326	0.171
No-Pin mm^2^	1.25 ± 0.80	0.09 ± 0.16	0.37 ± 0.36	1.72 ± 1.02
Pin mm^2^	1.28 ± 0.82	0.73 ± 0.66	2.35 ± 2.08	4.35 ± 3.38
*p* value	0.966	0.094	0.083	0.171

## Data Availability

The original contributions presented in the study are included in the article, further inquiries can be directed to the corresponding author.
